# Unique Pathogen Peptidomes Facilitate Pathogen-Specific Selection and Specialization of MHC Alleles

**DOI:** 10.1093/molbev/msab176

**Published:** 2021-06-10

**Authors:** Onur Özer, Tobias L Lenz

**Affiliations:** 1 Research Group for Evolutionary Immunogenomics, Max Planck Institute for Evolutionary Biology, Plön, Germany; 2 Research Unit for Evolutionary Immunogenomics, Department of Biology, Universität Hamburg, Hamburg, Germany

**Keywords:** HLA/MHC genes, human leukocyte antigen, pathogen-mediated balancing selection, pathogen peptidome, antigen binding

## Abstract

A key component of pathogen-specific adaptive immunity in vertebrates is the presentation of pathogen-derived antigenic peptides by major histocompatibility complex (MHC) molecules. The excessive polymorphism observed at MHC genes is widely presumed to result from the need to recognize diverse pathogens, a process called pathogen-driven balancing selection. This process assumes that pathogens differ in their peptidomes—the pool of short peptides derived from the pathogen’s proteome—so that different pathogens select for different MHC variants with distinct peptide-binding properties. Here, we tested this assumption in a comprehensive data set of 51.9 Mio peptides, derived from the peptidomes of 36 representative human pathogens. Strikingly, we found that 39.7% of the 630 pairwise comparisons among pathogens yielded not a single shared peptide and only 1.8% of pathogen pairs shared more than 1% of their peptides. Indeed, 98.8% of all peptides were unique to a single pathogen species. Using computational binding prediction to characterize the binding specificities of 321 common human MHC class-I variants, we investigated quantitative differences among MHC variants with regard to binding peptides from distinct pathogens. Our analysis showed signatures of specialization toward specific pathogens especially by MHC variants with narrow peptide-binding repertoires. This supports the hypothesis that such fastidious MHC variants might be maintained in the population because they provide an advantage against particular pathogens. Overall, our results establish a key selection factor for the excessive allelic diversity at MHC genes observed in natural populations and illuminate the evolution of variable peptide-binding repertoires among MHC variants.

## Introduction

Major histocompatibility complex (MHC) molecules mediate the adaptive immune response in jawed vertebrates by binding to short peptides and presenting them on the cell surface. These peptide:MHC complexes on the cell surface are continuously surveyed by T lymphocytes to detect the presence of infectious agents. The excessively high number of alleles at the classical MHC genes, in humans for instance with several thousand for each MHC class-I locus (Robinson et al. 2020), is considered to be maintained by pathogen-mediated balancing selection (Bodmer 1972; Hedrick and Thomson 1983; Radwan et al. 2020). In support of this hypothesis, most of the polymorphism within the MHC genes is observed in the residues forming the peptide-binding region of the MHC molecule, that is, the region that interacts with the presented peptide (Parham 1988; Robinson et al. 2017). The rate of nonsynonymous variation is much higher in the peptide-binding region compared with the rest of the MHC genes (Hughes and Nei 1988) and many of these variants are observed at intermediate frequencies (Brandt et al. 2018).

Three distinct yet not mutually exclusive mechanisms of pathogen-mediated balancing selection, namely heterozygote advantage, negative frequency-dependent selection, and fluctuating selection, have been proposed relatively early on and have been analyzed in a trove of different studies in various species over the last decades (Apanius et al. 1997; Spurgin and Richardson 2010; Radwan et al. 2020). According to the heterozygote advantage hypothesis, individuals with heterozygous MHC genotype present a higher coverage of peptides and, consequently, are able to mount an immune response against a larger range of pathogens compared with homozygotes (Doherty and Zinkernagel 1975; Hughes and Nei 1988). The heterozygote advantage hypothesis is further extended by a divergent allele advantage model, which relies on the assumption that MHC alleles that are divergent at the sequence level would have a low overlap in their peptide repertoires (Wakeland et al. 1990). Several theoretical as well as computational studies have supported the role of the divergent allele advantage model in maintaining allelic diversity (Lenz 2011; Pierini and Lenz 2018; Stefan et al. 2019). The negative frequency-dependent selection hypothesis assumes that most of the pathogens evolve much faster than their hosts and adapt to evade recognition by the most common MHC alleles (Bodmer 1972). Such adaptation provides a selective advantage to rare or novel MHC alleles and leads to cyclic fluctuations in allele frequencies (Ejsmond and Radwan 2015; Lenz 2018; Phillips et al. 2018). Finally, fluctuating selection on distinct MHC alleles is expected if the prevalence or selection pressure of pathogens changes over time or across geographical locations (Hedrick 2002; Dunn et al. 2010; dos Santos Francisco et al. 2015). All three mechanisms are based on two main assumptions: 1) that each pathogen challenges the MHC-based immune system in a different way, and 2) that MHC variants differ in the repertoire of presented peptides (from here on, the term 'variant' refers to distinct MHC/HLA molecule variants, encoded by distinct classical HLA alleles at 2^nd^-field resolution) .

**Fig. 1.**
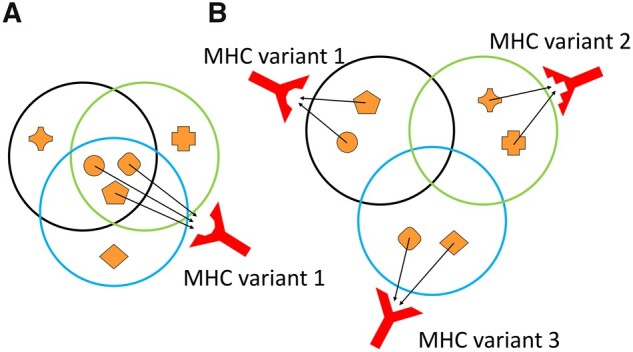
Conceptual representation of selection of different MHC variants depending on the peptidome compositions of pathogens. Empty circles represent peptidomes of three different pathogens, whereas orange icons represent representative antigens. If peptide sharing among pathogens is extensive (*A*), a single MHC variant would be enough to mount an immune response against all pathogens. If the peptide sharing among pathogens is very low (*B*), different MHC variants would be required for effective overall pathogen control.

The first assumption, that each pathogen challenges the adaptive immune system in a novel way, assumes that pathogens exhibit distinct antigenic peptide composition (fig. 1). Although the extent of antigenic diversity among pathogens is crucial for our understanding of the evolution of MHC genes, it has been analyzed only in few studies with a limited number of pathogen species, and mainly in the context of self/nonself overlap of peptides (Burroughs et al. 2004; Calis et al. 2012). So, although this first assumption appears widely accepted, systematic empirical evidence supporting this assumption is still lacking. The second assumption, that MHC variants differ in their repertoire of presented peptides, is empirically well supported (Sidney et al. 2008; Schellens et al. 2015; Pierini and Lenz 2018) and so is the fact that different MHC variants are associated with different infectious diseases (Trowsdale 2011; Tian et al. 2017; Sanchez-Mazas 2020). However, how exactly the variant-specific peptide repertoire leads to the variant’s effect on disease risk is still a matter of intense research (Radwan et al. 2020). In fact, the differential ability of MHC variants to trigger an immune response against specific pathogens can be determined by both quantitative (binding many or few peptides of a given peptide pool) and qualitative (binding or not binding of specific peptides) differences among variants. Croft et al. (2019) have shown that up to 80% of viral peptides that are presented on MHC class-I molecules can be immunogenic in mice. This suggests that selection might act more on quantitative differences among MHC variants, that is, binding more peptides from a specific pathogen is advantageous. In line with that idea, Arora et al. (2020) have shown that in HIV-infected individuals, the viral load is negatively correlated with the number of HIV peptides that are predicted to be presented by an individual’s HLA-B variants. On the other hand, they also showed that the presence of a specific HLA-B variant (B*57:01) alone provided a stronger protective effect than the protective effect achieved by merely binding many peptides per se (Arora et al. 2019). Indeed, several studies report that HLA-B restricted T-cell responses in HIV-1 infected individuals with slow disease progression tend to target conserved regions of the HIV-1 (Gillespie et al. 2006; Costa et al. 2010; Kunwar et al. 2013). Similar observations on other pathogens such as hepatitis C virus (Rao et al. 2015) or influenza (Eickhoff et al. 2019) indicate that not only the quantity but also the quality of peptides presented on MHC molecules affects disease outcome. It thus remains an open question how quantitative and qualitative differences in peptide binding among MHC variants contribute to their disease association, and thus to which extent each of these properties are the target of pathogen-mediated selection.

Intriguingly, recent studies have revealed that there is indeed substantial quantitative variation in the size of the bound peptide repertoire (i.e., total number of bound peptides, hereafter referred to as “promiscuity”) among MHC variants (Paul et al. 2013; Chappell et al. 2015). Along this promiscuity scale, promiscuous MHC variants bind a wide range of peptides, whereas fastidious variants are much more stringent in peptide binding and exhibit narrow repertoires. Manczinger et al. (2019) showed that the frequency of promiscuous MHC class-II variants is positively correlated with the pathogen-richness across countries, possibly because more promiscuous MHC variants provide an advantage by facilitating recognition of more pathogens. However, it is yet to be determined how fastidious MHC variants, which present a smaller peptide repertoire, are maintained in populations. One intriguing hypothesis, proposed by Kaufman (2018), postulates that fastidious variants may have a selective advantage if they are specialized against particular pathogens, especially if the immunodominant peptides are highly conserved (Schneidewind et al. 2007; Miura et al. 2009). Previous studies focusing on modeling approaches have shown that such specialization against specific pathogens may contribute to maintenance of high polymorphism at the MHC locus (Hedrick 2002). Although the number of experimental assays investigating peptide-MHC interactions increased rapidly in the last years (Vita et al. 2019), most of the empirical evidence is still focused on a few very common MHC variants and specific pathogen proteins. Therefore, it remains challenging to empirically test hypotheses on MHC specialization across a wide range of variants and pathogens. Computational approaches for the prediction of peptide-binding by MHC variants fill this gap to some extent (Peters et al. 2020). In fact, recent advances in prediction algorithms allow relatively accurate characterization of binding specificities even for MHC variants for which there is no empirical data available (Reynisson et al. 2020).

Here, we analyzed the potential antigenic diversity of 36 representative human pathogens and show that each pathogen harbors a distinct peptide pool, with only few peptides shared among pathogens. We then investigated how this antigenic diversity is reflected in the peptide-binding properties among human MHC variants. We characterized the variant-specific repertoire of bound peptides for a set of 321 common HLA class-I variants using computational binding prediction. Our results revealed an extensive variation in peptide-binding promiscuity among MHC variants as well as signatures of specialization mainly for fastidious variants.

## Results

### Human Pathogen Peptidome

**Fig. 2.**
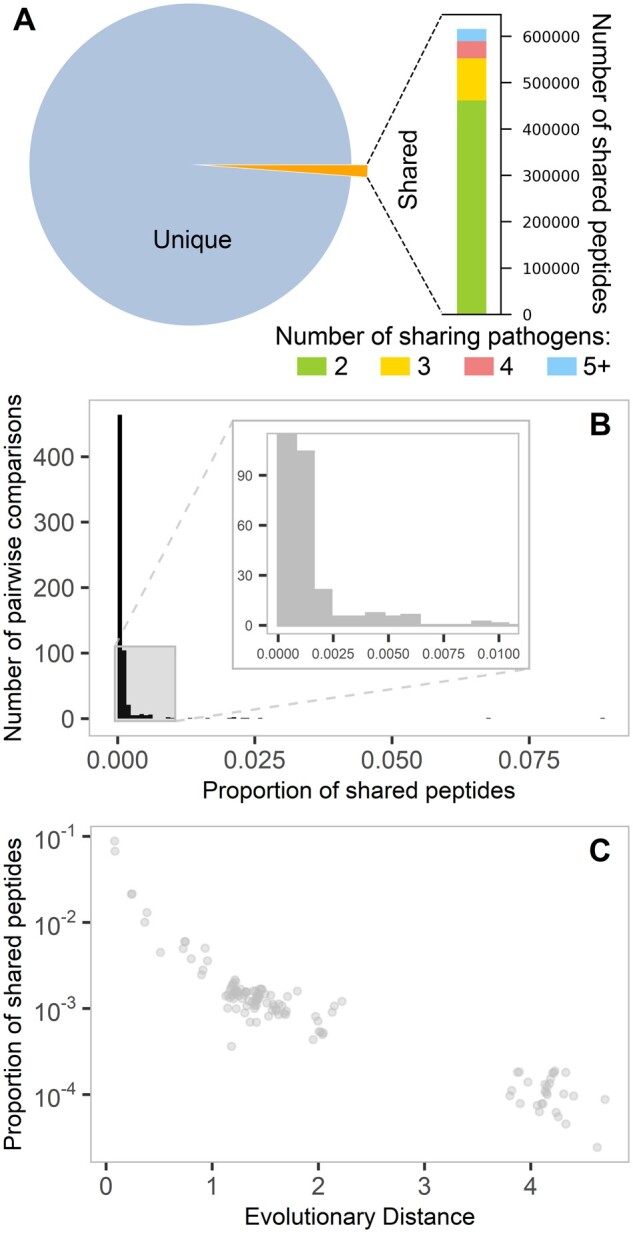
Peptide sharing among human pathogens. (*A*) Shared nine-mer peptides constitute a very small part of the human pathogen peptidome. The pie chart represents the proportions of shared (*N* = 615,904) and unique (*N* = 51,861,826) nine-mer peptides, whereas the bar chart shows the extent of sharing across pathogen species for all shared peptides. (*B*) Distribution of the pairwise peptide sharing among all pathogens (*N* = 36). The fraction of shared peptides out of all peptides bound by either pathogen is shown for all pathogen pairs (*N* = 630). (*C*) Pairwise peptide sharing among a subset of pathogens (*N* = 16) decreases with increased evolutionary distance. Evolutionary distance between pairs of pathogens was calculated as tip-to-tip distances within the tree of life. See supplementary table S1, Supplementary Material online, for organisms used in the analysis.

Complete proteomes of a representative set of diverse human pathogen species, including viruses (*N* = 10), bacteria (*N* = 19), and eukaryotic parasites (*N* = 7, supplementary table S1, Supplementary Material online) were divided into all possible nine amino acid long peptides, shortly nine-mers, by employing a sliding window approach with a step size of one amino acid, resulting in 51,861,826 nine-mers reflecting a broad representation of the human pathogen peptidome. The number of peptides per pathogen species ranged from 1,760 to 11,405,499 (median: 546,629.5, supplementary table S1, Supplementary Material online). Of all the nine-mers, 98.8% were unique to the given pathogen from which they originated, thus only 1.2% were shared among two or more pathogens (fig. 2*A*). Pairwise comparisons of peptide sharing among pathogens revealed that pathogens on average shared only a tiny fraction of their peptides (median: 0.005%; range: 0–8.8%), with 39.7% of the 630 pathogen pairs showing no shared peptide at all and only 11 pairs (1.8%) sharing more than 1% of their peptides (fig. 2*B*). For some pathogens with large peptidome sizes, this can amount to large absolute numbers of peptides (supplementary fig. S1, Supplementary Material online), even though it remains negligible in relative terms. In a subset of bacterial (*N* = 14) and eukaryotic (*N* = 2) pathogens, for which evolutionary divergence information was available (supplementary table S1, Supplementary Material online), peptide sharing was found to be negatively correlated with the evolutionary distance between pathogens (Kendall’s tau = −0.7, *P* < 0.001) (fig. 2*C*), suggesting a dominant role for sequence homology based on phylogenetic relatedness as a major determinant of peptide sharing.

### Peptide-Binding Promiscuity of HLA Variants

Having established that each pathogen is likely to challenge the adaptive immune system with a distinct set of peptides (fig. 1*B*), we next sought to investigate whether and how HLA molecules have adapted to this extreme peptidome diversity. Here, we use the term “HLA variant” to denote distinct variants of the classical HLA molecules that are encoded by a distinct HLA allele at second field resolution. In other words, each HLA variant corresponds to an HLA molecule with a distinct amino acid sequence. Prior studies have shown that the peptide-binding promiscuity (i.e., size of the repertoire of bound peptides) varies markedly among HLA variants (Paul et al. 2013; Chappell et al. 2015), raising the question of how HLA alleles that encode molecule variants that bind only few peptides are maintained in the population. Kaufman (2018) suggested that such fastidious HLA variants might provide an advantage through specialization toward particular pathogens. In the light of the distinct pathogen peptidomes shown above, this possibility appears plausible.

In order to investigate this hypothesis in more detail, comprehensive data about the peptide repertoires of a large number of different HLA variants are required. The optimal data for such an analysis would be derived from in vitro HLA:peptide binding or peptide elution assays, but such empirical data are so far only available for a very limited set of nonrandomly selected HLA variants and peptide repertoires. Another possibility for approaching the variation in peptide-binding promiscuity is to utilize computational peptide-binding prediction algorithms. These machine-learning algorithms are developed in the context of vaccine development and cancer immunotherapy and have been improved in accuracy over the past decade (Schirle et al. 2001; Paul et al. 2020). They are now well established and used in a wide range of contexts, including evolutionary genetic studies of the MHC (Lenz 2011; Buhler et al. 2016; Pierini and Lenz 2018; Manczinger et al. 2019; Arora et al. 2020). These algorithms are still imperfect in accurately predicting the antigenicity of specific peptides, however, they perform relatively well in predicting overall repertoires of bound peptides for a given HLA variant (Paul et al. 2020). For the present analysis of variant-specific overall peptide repertoire sizes, we focus on HLA class I variants, because their binding motifs are more clearly defined and computational binding prediction is considered to be more accurate for HLA class I (Reynisson et al. 2020). We thus rely on one of the most established HLA:peptide-binding prediction algorithm in order to study all classical HLA class-I alleles that are classified as “common” in the CIWD alleles catalogue (Hurley et al. 2020). Binding affinities between all unique nine-mer peptides and the selected HLA class-I variants were computationally predicted. Promiscuity of an HLA variant was defined as the fraction of peptides bound by the variant (with an affinity below a defined threshold) out of the complete set of unique peptides (*n* = 51,861,826 nine-mers). Promiscuity values were highly correlated between 50 nM (strong binders) and 500 nM (strong and weak binders) thresholds (Kendall’s tau = 0.82, *P* < 0.001). Therefore, for the rest of the analysis, a threshold of 500 nM was used. Variants having the exact same binding prediction results and the same first field number as another variant with a lower second field number (representing highly related alleles with negligible sequence difference) were removed, resulting in 82 HLA-A, 180 HLA-B, and 59 HLA-C variants for subsequent analyses. The correspondence between the computational and experimental promiscuity values was tested for a subset of HLA-A (*N* = 19) and HLA-B (*N* = 15) variants, for which experimental data were available from the IEDB database (Vita et al. 2019). A moderate correlation was observed for both HLA-A (Kendall’s tau = 0.51, *P* = 0.002) and HLA-B variants, although the latter was not statistically significant (Kendall’s tau = 0.37, *P* = 0.054, supplementary fig. S2, Supplementary Material online), possibly owing to the small number of variants and the limited and nonrandom collection of peptides in the IEDB data.

Using the newly obtained information of predicted HLA variant-specific peptide binding, we first reanalyzed the sharing of peptides among pathogens. Peptides that were predicted to be bound by the same set of HLA variants (out of all HLA variants) were merged so that each merged peptide group represents all peptides that are equivalent from the HLA perspective (see Materials and Methods). Peptide sharing based on the merged peptide groups (*N* = 4,157,475) showed that still 85.6% of groups were unique to a specific pathogen, with the rest shared by two or more pathogens (supplementary fig. S3, Supplementary Material online).

We then used the HLA:peptide binding data to investigate the variation in peptide repertoire sizes (i.e., promiscuity) among HLA class I variants. Promiscuity of individual HLA variants varied greatly within and between loci (supplementary fig. S4, Supplementary Material online). Although both promiscuous (i.e., with large peptide repertoire) and fastidious (i.e., with small peptide repertoire) variants are observed at all loci, HLA-B and HLA-C variants appear to have narrower peptide repertoires when compared with the HLA-A variants (pairwise Wilcoxon rank sum test, HLA-A and HLA-B: *P* < 0.001, HLA-A and HLA-C: *P* = 0.005). The difference in promiscuity between HLA-A and HLA-B variants was confirmed by two experimental data sets, the IEDB data set (Wilcoxon rank sum test, *P* = 0.026) and a data set curated by Abelin et al. (2017) (Wilcoxon rank sum test, *P* = 0.02). Analysis of promiscuity in a phylogenetic context showed that large differences in the peptide repertoire size of HLA variants can evolve quickly within all loci as closely related variants can differ markedly in promiscuity (supplementary fig. S5, Supplementary Material online).

### Specialization of HLA Variants in Peptide Binding

**Fig. 3.**
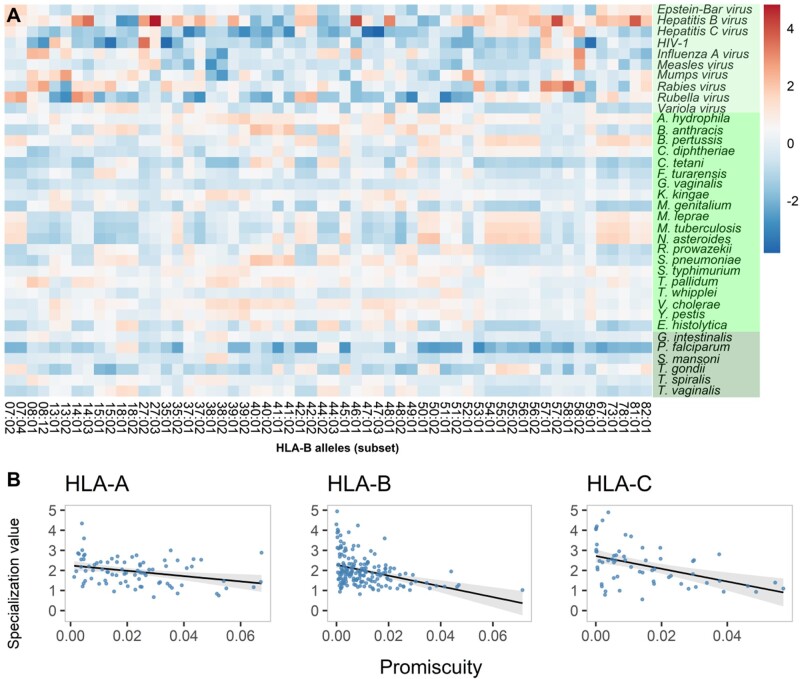
(*A*) Standardized proportions of bound peptides from distinct pathogens varies greatly among HLA-B variants. Each cell represents the proportion of bound peptides by the HLA variant (on horizontal axis) from the corresponding pathogen (on vertical axis). Proportions are standardized within each variant to make comparisons across variants possible. Dark red color indicates high specialization, white no specialization, and dark blue indicates lower than average binding of a pathogen’s peptides. Green shading on vertical axis labels indicates different pathogen groups (light green; viruses, green; bacteria, dark green; eukaryotes). In this plot, only a subset of HLA-B variants were included for better visualization. The subset was selected such that a maximum of two variants with the same first field number and smallest second field number were included (e.g., only B*15:01 and B*15:02 of all the variants of the B*15 lineage). The patterns are similar across all variants of all three HLA loci. For the complete set of HLA-A, HLA-B, and HLA-C variants, see supplementary figure S7, Supplementary Material online. (*B*) Specialization is negatively correlated with promiscuity for all HLA class I loci. Each dot represents an HLA variant of the given HLA gene, shown separately for *HLA-A* (*n* = 82), *HLA-B* (*n* = 180), and *HLA-C* (*n* = 59). Specialization was calculated for each variant as the difference between the maximum and the median values of standardized proportions of bound peptides. Promiscuity was calculated for each HLA variant as the fraction of the bound peptides among the complete data set of 51.9 Mio peptides. Linear regression line is shown in black and 95% CI around the line in gray.

The observed large differences in promiscuity among HLA class-I variants confirmed previous empirical studies on more limited sets of variants (Paul et al. 2013; Chappell et al. 2015). However, this observation raises the question of whether the variation in promiscuity is a random byproduct of sequence evolution of the underlying HLA alleles, and also emphasizes the puzzle how fastidious variants are maintained in the population. According to the hypothesis by Jim Kaufman (Chappell et al. 2015; Kaufman 2018), promiscuous variants might act as generalists, providing protection from a large set of common pathogens, whereas fastidious variants may be specialized against one or few pathogens. Specialization may confer a selective advantage to fastidious variants especially in times of outbreaks or persistent high pathogen pressure by these specific pathogens. In order to investigate potential specialization and test this hypothesis in a quantitative way, we calculated for each variant the normalized fraction of bound peptides from each pathogen. Overall, the peptide-binding values (standardized for pathogen proteome size and HLA variant promiscuity—see Materials and Methods) for each HLA class-I locus were normally distributed (supplementary fig. S6, Supplementary Material online). Yet, within each locus, there are HLA variant–pathogen pairs with distinct associations, potentially indicating nonrandom relationships (fig. 3*A* and supplementary fig. S7, Supplementary Material online). In order to analyze these peptide-binding patterns in more detail, a specialization value was calculated for each HLA class-I variant that reflects the relative difference between the variant’s ability to bind peptides of its best-covered pathogen compared with all pathogens. A high specialization score indicates that the variant binds particularly many peptides from its best-covered pathogen, compared with the number of peptides it generally binds across all pathogens. Intriguingly, this specialization value was negatively correlated with the promiscuity of variants for all HLA class-I loci (Kendall correlation, HLA-A: tau = −0.22, *P* = 0.003; HLA-B: tau = −0.28, *P* < 0.001; HLA-C: tau = −0.33, *P* < 0.001; fig. 3*B*). In other words, fastidious variants tend to have higher specialization values than promiscuous variants. Stronger correlations were observed using a 50 nM threshold that includes only strong binders (Kendall correlation, HLA-A: tau = −0.37, *P* < 0.001; HLA-B: tau = −0.37, *P* < 0.001; HLA-C: tau = −0.62, *P* < 0.001). Although we analyzed a comprehensive set of all pathogens here, we expect that this relationship would also hold only among strictly intracellular pathogens, given that the viruses in this data set generally exhibited the most extreme values of specialization, and that there are intracellular pathogens also among the other pathogen groups. In order to rule out the possibility that the observed negative correlation between promiscuity and specialization is driven by general variation related to both the overall differences in peptide repertoire sizes among variants and differences in peptidome sizes of pathogens, a simulated version of the binding data for all three HLA loci was generated for each variant by using the promiscuity values of the variants as probabilities of binding a given peptide from a given pathogen (see Materials and Methods). This simulation mimicked the observed data distribution and variation within and among variants except for any potential preference toward specific pathogens. The simulated data were then analyzed in the same way as the real data. No correlation between promiscuity and specialization was observed in the simulated data (Kendall correlation, HLA-A: tau = 0.05, *P* = 0.54; HLA-B: tau = −0.02, *P* = 0.65; HLA-C: tau = 0.01, *P* = 0.93; supplementary fig. S8, Supplementary Material online).

**Fig. 4.**
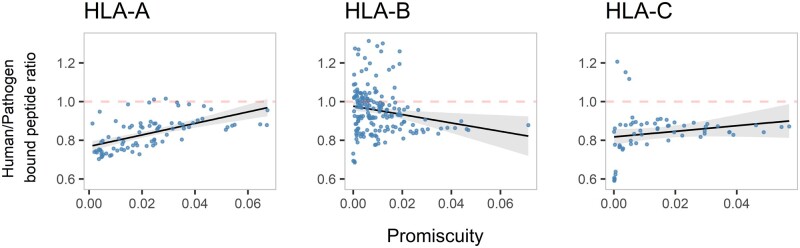
Self to nonself binding ratios as a function of variant promiscuity. Each dot represents an HLA variant of the given HLA gene, shown separately for *HLA-A* (*n* = 82), *HLA-B* (*n* = 180), and *HLA-C* (*n* = 59). Dashed red line indicates a 1/1 ratio, that is, an equal tendency to bind either human or pathogen peptides. Promiscuity was calculated for each HLA variant as the fraction of the bound peptides among the complete data set of 51.9 Mio peptides. Linear regression line is shown in black and 95% CI around the line in gray.

Another interesting question regarding the evolution of MHC variants and specifically the specialization toward specific peptide repertoires concerns the preferential binding of foreign (i.e., pathogen derived) and self-peptides. It was previously shown based on predicted peptide-binding data that some MHC class-I molecules, specifically HLA-A variants, preferentially present pathogen-derived peptides over self-peptides (Rao et al. 2009; Calis et al. 2010). Following that observation, we therefore used our approach to investigate the relationship between an MHC variant’s promiscuity and binding of foreign over self-peptides. The ratio of self-binding fraction (fraction of bound self-peptides over all self-peptides) to foreign-binding fraction was used as a proxy for a variant’s preference toward self- or foreign-derived peptides (fig. 4). In accordance with the results of Rao et al., (2009) all HLA-A variants were found to have self to foreign-binding fractions lower than or very close to one, indicating a binding preference toward foreign peptides. Moreover, we observed a significant positive correlation between the self to foreign-binding ratio and promiscuity (Kendall correlation, tau = 0.47, *P* < 0.001), indicating that fastidious HLA-A variants tend to have a significantly higher specificity toward foreign peptides than promiscuous HLA-A variants. A similar positive correlation was also observed for the *HLA-C* locus (Kendall correlation, tau = 0.22, *P* = 0.013) and with the exception of three fastidious HLA-C*01 variants, all HLA-C variants had self to foreign-binding fractions lower than one. In contrast to *HLA-A* and *HLA-C* loci, a weak negative correlation between the self to foreign-binding ratio and promiscuity was observed for the *HLA-B* locus (Kendall correlation, tau = −0.14, *P* = 0.006). Interestingly, promiscuous HLA-B variants consistently had self to foreign-binding ratio of lower than one, whereas both high and low ratios were observed in fastidious variants (fig. 4).

Despite the low proportions of shared peptides among pathogens (fig. 2*B*) the absolute numbers of shared peptides can amount up to several thousand among pathogens with large peptidome size (supplementary fig. S1, Supplementary Material online). Therefore, we also investigated whether promiscuous and fastidious variants differ in binding to peptides shared by three or more pathogens, as a specialization of fastidious variants toward shared peptides would provide a selective advantage by facilitating simultaneous recognition of multiple pathogens. For this, the ratio of the fraction of bound unique peptides to the fraction of bound shared peptides (by three or more pathogens) were compared between the most promiscuous (top 25%) variants and the least promiscuous (bottom 25%, i.e., the most fastidious) variants within each HLA locus. No significant differences were observed for HLA-B and HLA-C, whereas fastidious HLA-A variants seem to bind more unique peptides than promiscuous ones (Wilcoxon rank sum test, *P* = 0.005; supplementary fig. S9, Supplementary Material online).

## Discussion

Our analysis reveals the vast diversity of the human pathogen peptidome and provides insights into how different peptide-binding properties of HLA molecules might have evolved to cope with this diversity. The analysis of peptidome diversity in a diverse set of pathogenic organisms showed that the overwhelming majority of the nine-mer peptides were unique to the pathogen from which they originate. The extreme diversity of peptides among pathogens strongly supports the assumption that the evolution of high allelic diversity of HLA genes is driven by the need for diverse antigen presentation. It also suggests that every pathogen has indeed a different selective effect on the HLA allele pool (fig. 1*B*) and thus provides the empirical basis for pathogen-by-allele interaction scenarios that underlie two of the most commonly assumed balancing selection mechanisms: negative frequency-dependent selection and fluctuating selection (Radwan et al. 2020). Our results most likely represent a lower limit of peptide sharing mainly because we consider the whole length of the nine-mers for the analysis in a set of diverse pathogens. Many peptides with a few amino acid differences can be considered as equivalents from the MHC perspective especially if the differences are among amino acids with similar chemical properties and outside of the anchor residues (Rammensee et al. 1999). However, even when taking a very conservative perspective of peptide sharing by merging peptides based on similarity in the set of HLA variants binding a given peptide, we still find the vast majority of merged peptides being unique to a given pathogen.

Several studies reported cross-reactive T-cell responses against closely related viruses (Weiskopf et al. 2013; Eickhoff et al. 2019) or bacteria (Abate et al. 2019) which is in line with our result of increased peptide sharing with smaller evolutionary divergence among pathogens. It should also be noted that even among highly unrelated pathogens, sharing of few but particularly immunogenic peptides could still lead to cross-reactivity in the host immune response. However, none of the HLA variants in our data set had a particular binding preference toward the peptides shared by two or more pathogens. Many of the shared peptides likely originate from proteins that are highly conserved across organisms, possibly including humans. Targeting of HLA variants toward shared peptides may be ineffective, if they are also shared by humans, because T-cells recognizing those specific peptide-HLA complexes would be eliminated during the thymic selection. Alternatively, if shared peptides are not enriched in any specific sequence motif compared with unique peptides, it becomes impossible for an HLA variant to specialize on shared peptides.

The observed highly distinct peptidome composition of pathogens provides a basis for the hypothesis that some HLA variants might be specialized against particular pathogens. In fact, the computational quantification of peptide binding by common HLA variants revealed substantial variation among different pathogens and HLA variants in the proportion of bound peptides (fig. 3*A* and supplementary fig. S7, Supplementary Material online). Moreover, applying a specialization metric to each HLA variant, we found that the variants having higher specialization scores tend to have narrower peptide-binding repertoires. This observation supports the hypothesis that specialization on one or a few pathogens might provide a selective advantage to fastidious alleles (Kaufman 2018).

One important question regarding our analysis is to what extent the specialization of an HLA variant toward a pathogen coincides with the protection from infection. Our specialization metric is based on comparing different pathogens with respect to the proportions of their complete peptides that are predicted to be bound by an HLA variant. Recently, Arora et al. (2019) showed that the protective effect of HLA-B alleles against HIV-1 viral load is positively correlated with the number of HIV-1 peptides that a given HLA variant is predicted to bind, an effect that was also observable at the genotype level where HIV-1-infected individuals whose HLA-B variants together were predicted to bind more HIV-1 peptides also exhibited a lower viral load and thus a slower progression toward AIDS (Arora et al. 2020). However, MHC-related determinants of disease outcome are more complex than the mere quantity of bound peptides. It was demonstrated for several pathogens such as HIV-1 (Borghans et al. 2007) and hepatitis-C virus (Rao et al. 2015) that HLA variants that are associated with effective disease control target conserved regions of the pathogen proteome (Hertz et al. 2011). Moreover, proteins that are expressed by a pathogen throughout the infection vary greatly due to sex-specific (Lasonder et al. 2016) or stage-specific (Lin et al. 2016) effects, thus also affecting the potential repertoires of presented peptides. Finally, peptides need to go through the steps of the antigen processing pathways, such as proteolytic cleavage or translocation into endoplasmic reticulum before being presented by HLA molecules (Yewdell et al. 2003; Blum et al. 2013). This suggests that only a subset of all possible peptides is actually presented by HLA molecules on the cell surface and some of those presented peptides may be more important than others. It was shown for a few HLA class I alleles that promiscuity is inversely correlated with the cell surface expression of the corresponding HLA molecules (Chappell et al. 2015). If such relationship holds true in general, persistent presentation of a few immunodominant pathogen peptides on the cell surface by fastidious HLA variants would indeed allow efficient pathogen control and such variants can truly be called specialists. Therefore, we do not expect to observe a perfect correlation between quantitative specialization of an HLA variant and pathogen control by individuals carrying that allele, and the specialization parameter presented in our analysis should thus be understood as a metric for an increased probability to present immunodominant peptides from a particular pathogen.

We have observed substantial variation among HLA variants in peptide-binding promiscuity, exceeding orders of magnitude, both within and between different HLA loci. The observed variation is not correlated with the allele divergence, indicating that promiscuous or fastidious HLA class-I variants may evolve quickly in response to varying pathogen pressure. The same conclusion was also reached by Manczinger et al. (2019) for HLA class II HLA-DRB1 variants, highlighting the role of promiscuity in pathogen-mediated selection for both HLA class-I and class-II loci. The median promiscuity level was significantly higher for variants of the *HLA-A* locus compared with *HLA-B* and *HLA-C* loci. Multiple studies on distinct properties of HLA class-I variants revealed differences among these loci, especially between HLA-A and HLA-B loci (Di et al. 2021). Prugnolle et al. (2005) reported that the positive correlation between pathogen richness and allelic diversity is much stronger for the *HLA-B* locus than the *HLA-A* locus. dos Santos Francisco et al. (2015) also noted a similar result that when the alleles were classified into supertypes, that is, allele groups with similar binding properties as determined by peptide-binding pockets, the effect of local adaptation is more evident for HLA-B supertypes. Based on these observations, it can be hypothesized that HLA-A variants tend to be more promiscuous generalists, whereas HLA-B variants tend to be more fastidious specialists that evolve quickly in response to varying pathogen pressures. This hypothesis is further supported by the finding of Hertz et al. (2011) that HLA-B variants effectively target conserved peptides of RNA viruses that are known to evolve very fast (Drake and Holland 1999). Furthermore, the *HLA-B* locus harbors the highest number of alleles among all HLA loci, which is in line with the idea that it most closely evolves with specific pathogens. It should be noted that such hypothesis does not exclude specialist HLA-A variants or generalist HLA-B variants as we also show that promiscuity can evolve very quickly (by few mutational steps).

Our data reveal an interesting relationship between promiscuity and self to nonself binding ratios especially for HLA-B. Promiscuous HLA-B variants clearly show a reduced preference toward human peptides, whereas no such preference was observed for fastidious HLA-B variants. These differences might be explained by T-cell selection in the thymus (Takaba and Takayanagi 2017). Chappell et al. (2015) hypothesized that low cell surface expression of promiscuous MHC variants might be an adaptation to prevent excessive depletion of T-cells that recognize a wide variety of self-peptides presented by promiscuous MHC molecules in the thymus. Following the same reasoning, promiscuous variants presenting fewer human self-peptides might be preferentially maintained as they lead to less T-cell depletion. Such depletion would not be problematic in the case of fastidious alleles due to the already small number of self-peptides presented, and no selection pressure for a decreased self-binding would be observed for fastidious variants. On the other hand, the correlation between promiscuity and self to nonself binding ratio for HLA-A was stronger, suggesting that even fastidious HLA-A variants might be under selection to bind fewer self-peptides. It is possible that different selection pressures acting on HLA-A and HLA-B loci lead to such differences. Hertz et al. (2011) noted an increased binding preference of HLA-A variants toward conserved human peptides compared with HLA-B variants. Whether the promiscuity has a role in such specialization toward human peptides needs to be investigated further.

In summary, we report here the first systematic characterization of the vast diversity among pathogen peptidomes and provide support for the hypothesis that fastidious MHC variants can be maintained in populations by virtue of specialization toward one or few pathogens. However, the relationship between peptide-binding promiscuity and specialization, and its role for MHC evolution is complex, and involves both qualitative and quantitative aspects of peptide binding. Our approach based on computational binding prediction can only partly capture this complexity, and focuses predominantly on the quantitative aspects of this relationship. Nevertheless, our results yield intriguing insights into pathogen diversity and the evolution of peptide promiscuity, and provide a basis for further research into the nuances of pathogen-mediated selection on the antigen-presentation pathways.

## Materials and Methods

### Selecting Pathogen Species and Peptide Data

The rationale behind the selection of pathogens used in this study was adopted from Pierini and Lenz (2018), following three main criteria: a global distribution of the pathogen, high mortality and/or morbidity (World Health Organization 2018), and an impact on the human history (Wolfe et al. 2007). A total of 36 pathogen species that likely had an important role in shaping the current diversity of human MHC genes were selected (Pierini and Lenz 2018), including ten viruses, 19 bacteria, and seven eukaryotic parasites. Reference proteomes of these pathogens as well as the reference proteome of Homo sapiens were downloaded from UniProt database (The UniProt Consortium 2019). For the specific species and accession numbers, see supplementary table S1, Supplementary Material online.

### Calculation of Peptide Sharing and Evolutionary Distance Values among Pathogens

Although the peptide-binding groove of different MHC class-I molecules can accommodate varying lengths of peptides, the median length of eluted peptides from MHC class I molecules is nine amino acids (Ritz et al. 2016; Abelin et al. 2017). The presented analyses are therefore based on nine-mer peptides. All possible nine-mers were obtained from pathogen proteins with a sliding window approach using a step size of one amino acid. Peptides containing ambiguous amino acid calls X, U, and B were removed (*N* = 11,457; 0.022% of total peptides) resulting in 51,861,826 nonredundant nine-mers. Peptide sharing among pathogens was analyzed with two separate approaches. With the first approach, sharing of peptides among pathogens were analyzed with respect to the complete sequence of each nine-mer. The second approach focuses only on the nine-mers bound by at least one HLA class-I variant (*N* = 19,222,466). Each nine-mer was assigned a code representing the HLA class-I variants that binds to it. Nine-mers having the same code were grouped together and considered as the same from the perspective of HLA molecules as they are bound by the same set of HLA class-I variants. In total 4,157,475 such groups (codes) were formed. Sharing of peptides among pathogens were analyzed with respect to these groups.

Pairwise peptide sharing among pathogens was calculated either as the proportion of shared peptides within the combined peptidome of pathogen pairs or as absolute number of shared peptides. Peptide sharing with respect to evolutionary divergence was analyzed among 14 bacteria and two eukaryotic parasites that were common between the data set used in this study and the tree of life (ToL) generated by Ciccarelli et al. (2006) (supplementary table S1, Supplementary Material online). Evolutionary divergence between pairs of pathogens was calculated as tip-to-tip distances within the tree of life by using the ape package in R (Paradis and Schliep 2019).

### HLA Variant Data

Three classical human MHC class-I genes (*HLA-A*, *-B*, and *-C*) were analyzed in this study. Although thousands of different alleles has been identified for each HLA loci (Robinson et al. 2020), most of these alleles are observed at very low frequencies or defined with limited documentation. Low frequency alleles are highly informative in some specific context such as organ transplantation (Kamoun et al. 2017) but their effect on recent human evolution is likely to be negligible (Robinson et al. 2017). In order to avoid biases that can be introduced by such alleles, two main criteria were applied on allele selection. Firstly, only alleles designated as “common” in the CIWD 3.0.0 catalogue were included in the analyses (Hurley et al. 2020). CIWD 3.0.0 catalogue classifies HLA alleles into categories based on their frequency. The “common” category of the CIWD catalogue covers the most frequent alleles in populations (those that are observed at a frequency of ≥0.01%). Secondly, in order to capture the functional diversity of MHC class-I genes while avoiding redundancy, P group designation of HLA alleles was used. Alleles within a P group have identical peptide-binding properties as they code for the same amino acid sequence across the antigen-binding domain (Marsh et al. 2010).

### Computational Prediction of Peptide-Binding

The set of potentially bound peptides for each given MHC variant in the study was estimated by using NetMHCpan(v4.1) (Reynisson et al. 2020). NetMHCpan is an established computational binding prediction algorithm that is trained on both experimental binding affinity and mass spectrometry-derived eluted ligand data. Based on the training data, it can predict the binding between any MHC molecule and peptide either as an affinity value or as a percentile rank score compared with a set of natural peptides. Although previous analysis indicates that percentile rank score performs better than the affinity score for identification of bound peptides, the percentile rank score assumes that all MHC variants bind the same number of peptides (Nielsen and Andreatta 2016). As the main aim of this study was to analyze differences in the size of the peptide repertoire of MHC variants, an affinity threshold of 50 and 500 nM were used to define bound peptides. The affinity threshold of 500 nM is widely considered as the limit of weak binding between an MHC variant and peptide, hence covering both strongly and weakly bound peptides (Paul et al. 2013), whereas the 50 nM threshold includes only strong binders. In order to avoid pseudoreplication, variants having the exact same binding prediction results and the same first field number were identified and 22 HLA-A, 18 HLA-B, and 12 HLA-C variants were removed by keeping only the variant having the smallest second field number. Promiscuity of an MHC variant was defined as the fraction of peptides bound by the variant from the complete set of unique peptides. In order to test whether the computational promiscuity values are in agreement with experimental results, data from the Immune Epitope Database (IEDB) were used (Vita et al. 2019). The IEDB is a collection of experimental data on T-cell and antibody responses against or MHC binding of epitopes. Complete data set of MHC ligand assays for HLA-A, -B and -C variants were downloaded on June 15, 2020. Assays for which the source organism of the peptide is either Homo sapiens or unidentified were removed. Furthermore, only assays with variants having second field (four-digit) or higher resolution were used. Finally, HLA variants having assay results for less than 1,000 different peptides were removed, leaving 19 HLA-A and 15 HLA-B variants for further analysis. No HLA-C variant met the criteria. Experimental promiscuity values were calculated as the fraction of positive binding assays among the total number of assays for each HLA variant. Kendall’s rank correlation test was used to analyze the relationship between experimental and computational promiscuity values of individual variants. Another data set of experimental binding data was also compiled using the number of peptides eluted by mass-spectrometry for nine HLA-A and six HLA-B variants from Abelin et al. (2017). However, due to the small number of variants, this data set was not used for correlations of individual variants and used only to calculate overall experimental promiscuity for HLA-A and HLA-B loci.

### Calculating Phylogenetic Distance between MHC Alleles

Complete protein sequences of HLA class-I alleles were downloaded on February 25, 2019 from IPD-IMGT/HLA Database (Robinson et al. 2020) and aligned with ClustalW software implemented in MEGA-X (Kumar et al. 2018). Positions that correspond to the peptide-binding region of HLA proteins were removed as these positions are under positive selection (Hughes and Hughes 1995) and also most likely involved in direct interaction with the peptide, thus defining the peptide-binding properties of the HLA variant. A phylogenetic tree was built separately for each HLA class-I locus using the maximum likelihood method with Jones–Taylor–Thornton substitution model to calculate amino acid distances and 1,000 bootstrap replicates to quantify support of nodes. Evolutionary distance between alleles was calculated as tip-to-tip distances in phylogenetic trees using ape package in R (Paradis and Schliep 2019).

### Calculating Pathogen Specialization of MHC Variants

Quantitative differences among HLA variants with regard to binding peptides from distinct pathogens were analyzed. In order to allow unbiased comparison of variants with different promiscuity levels, fractions of bound peptides from each pathogen were standardized by converting them to *z* scores within each variant. Without this normalization, variants that are more promiscuous would automatically have a higher variance of their relative peptide-binding values among the different pathogens, which would bias the specialization analysis. For each variant, a specialization value was then calculated as the difference between the maximum and the median *z* score. The rationale here is that a variant that is specialized to bind peptides of a specific pathogen particularly well should show a particularly high difference between the fraction of bound peptides from this pathogen and the fraction of all other pathogens (reflected by the median). A potential correlation between the specialization values and the promiscuity levels of the variants was tested using Kendall’s rank correlation. In order to verify that the obtained results were not driven by random fluctuations or any methodological bias in the binding data, simulations were performed. For these simulations, an HLA variant’s overall promiscuity level (fraction of peptides bound from the total number of peptides) is used as its probability of binding a peptide from a given pathogen. This probability was then used to randomly sample peptides from each pathogen peptidome and thus simulate the fraction of bound peptides from each pathogen under a no-specialization scenario. This Monte Carlo simulation approach was applied to all HLA loci separately. The difference to the real data was only that the fraction of peptide bound from each pathogen now reflected the overall promiscuity of the allele and not the pathogen-specific promiscuity. By using the same scaling approach, specialization value calculations and correlation test were then also applied to the simulated data. 

## Supplementary Material

Supplementary data are available at *Molecular Biology and Evolution* online.

## Supplementary Material

msab176_Supplementary_Data
