# Supplementary material for: Unique Pathogen Peptidomes Facilitate Pathogen-Specific Selection and Specialization of MHC Alleles
Source: Mol Biol Evol. 2021 Jun 10;38(10):4376–87. doi: 10.1093/molbev/msab176 (PMC8476153; doi:10.1093/molbev/msab176)
Supplement: msab176_Supplementary_Data [file msab176_supplementary_data.pdf]

Supplementary Material for

Unique pathogen peptidomes facilitate pathogen-specific selection and specialization of MHC alleles

Onur Özer[1,2] & Tobias L. Lenz[1,2,*]

Affiliations:

[1] Research Group for Evolutionary Immunogenomics, Max Planck Institute for Evolutionary Biology, 24306 Plön, Germany

[2] Research Unit for Evolutionary Immunogenomics, Department of Biology, Universität Hamburg, 20146 Hamburg, Germany

* Corresponding author: Tobias L Lenz (lenz@post.harvard.edu)

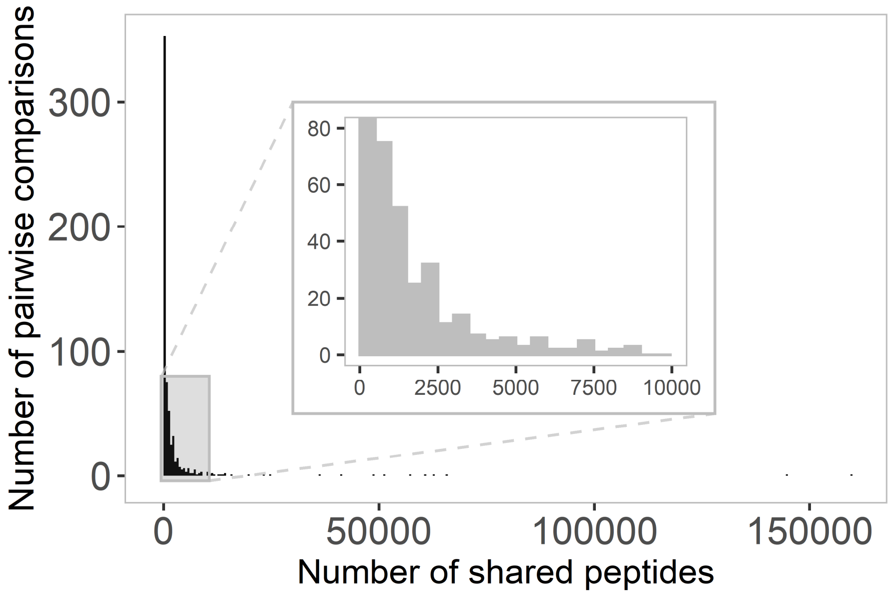

Figure S1. Distribution of the number of shared peptides among all pairs of pathogens (N=630). For each pathogen pair, shared peptides represent the overlap between both pathogen peptidomes.

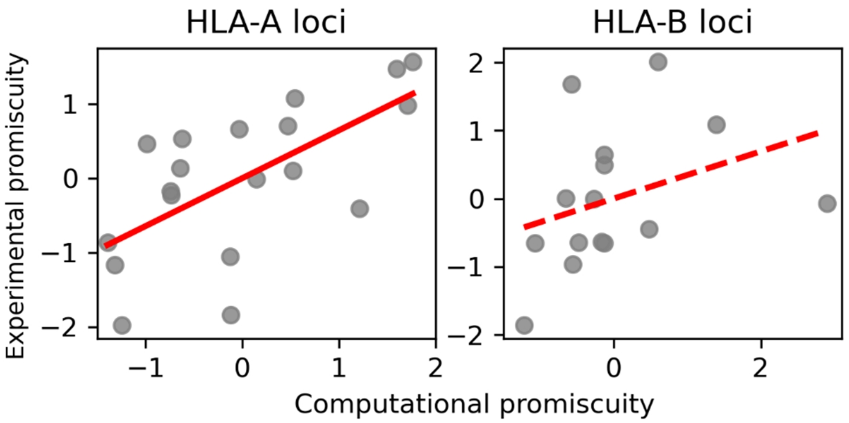

Figure S2. Experimental and computational promiscuity values are correlated for HLA-A (Kendall's tau = 0.51, p = 0.002) and HLA-B (Kendall's tau = 0.37, p = 0.054) loci. Each dot represents HLA-A (N = 19) and HLA-B (N = 15) variants for which sufficient experimental data is available on the Immune Epitope Database. Computational promiscuity was calculated as the fraction of the bound peptides among the complete dataset of 51.9 Mio peptides. Experimental promiscuity was calculated based on the data from the Immune Epitope Database as the fraction of positive binding assays among the total number of assays for each HLA allele. Computational and experimental promiscuity values were normalized for comparison. Solid red line represents significant correlation while dashed line represents positive trend. The HLA alleles included in the analysis are A*01:01, A*02:01, A*02:02, A*02:03, A*02:06, A*03:01, A*11:01, A*23:01, A*24:02, A*24:03, A*26:01, A*30:01, A*30:02, A*31:01, A*33:01, A*68:01, A*68:02, A*69:01, A*80:01, B*07:02, B*08:01, B*15:01, B*15:17, B*18:01, B*27:05, B*35:01, B*39:01, B*40:01, B*44:02, B*46:01, B*51:01, B*53:01, B*57:01, B*58:01.

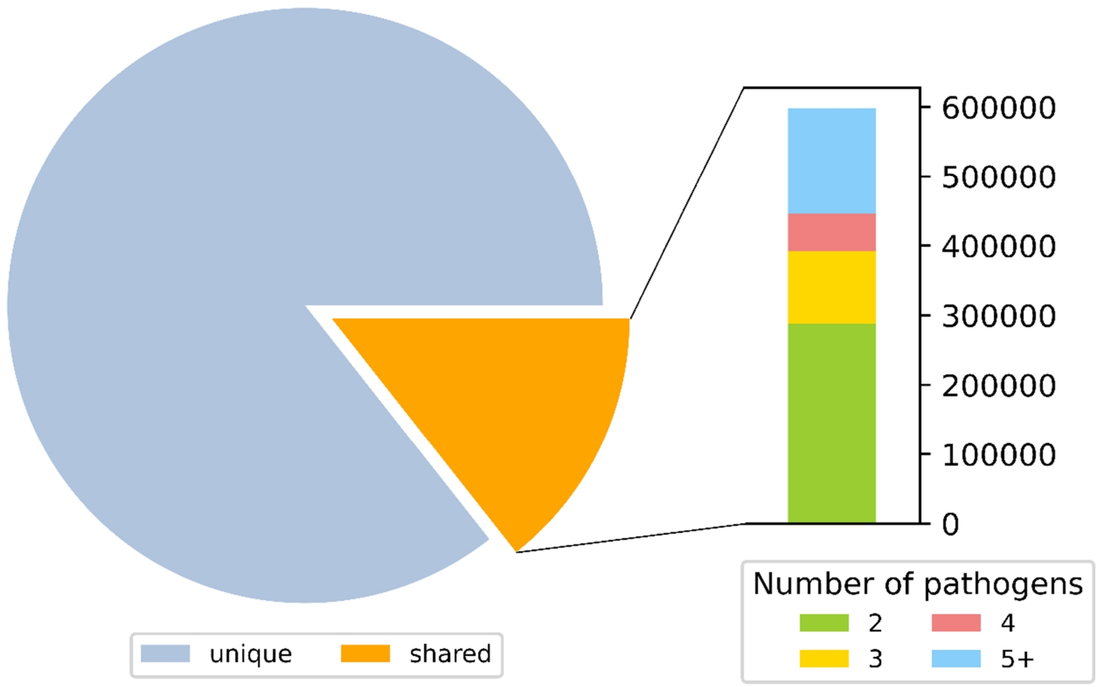

Figure S3. Peptide sharing among human pathogens based on groups of peptides that are bound by same set of HLA alleles. A total of 4,157,475 groups were analyzed and only 14.4% of these groups were shared among pathogens. The pie chart represents the proportions of shared (N = 597,700) and unique (N = 3,559,775) groups of peptides while the bar chart shows the extent of sharing across pathogen species for all shared peptide groups.

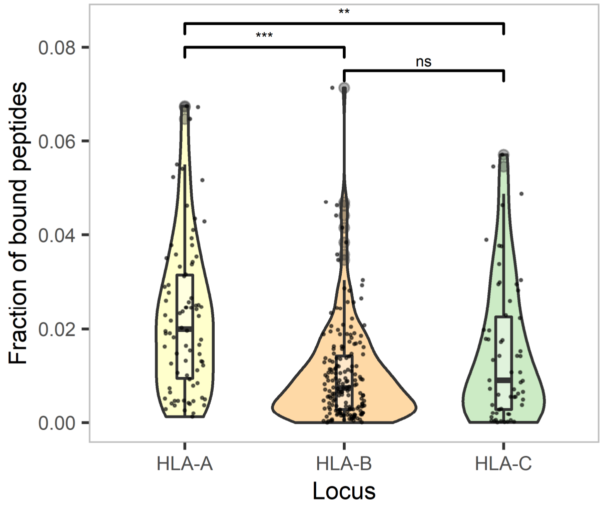

Figure S4. Variation of promiscuity within and among MHC loci. The fraction of bound peptides out of the complete set of unique peptides (N = 51,861,826 nine-mers) is shown for common variants of the three HLA class I loci (HLA-A; n = 82, HLA-B; n = 180 and HLA-C; n = 59). Each dot represents an HLA variant. Upper and lower edges of boxes correspond to the first and the third quartiles of the data while whiskers extend up to the data at most 1.5 IQR away from the edges of the box. Statistical significance from Wilcoxon rank sum test is indicated: *** - $p<0.001$, ** - $p<0.01$, ns - $p>0.05$.

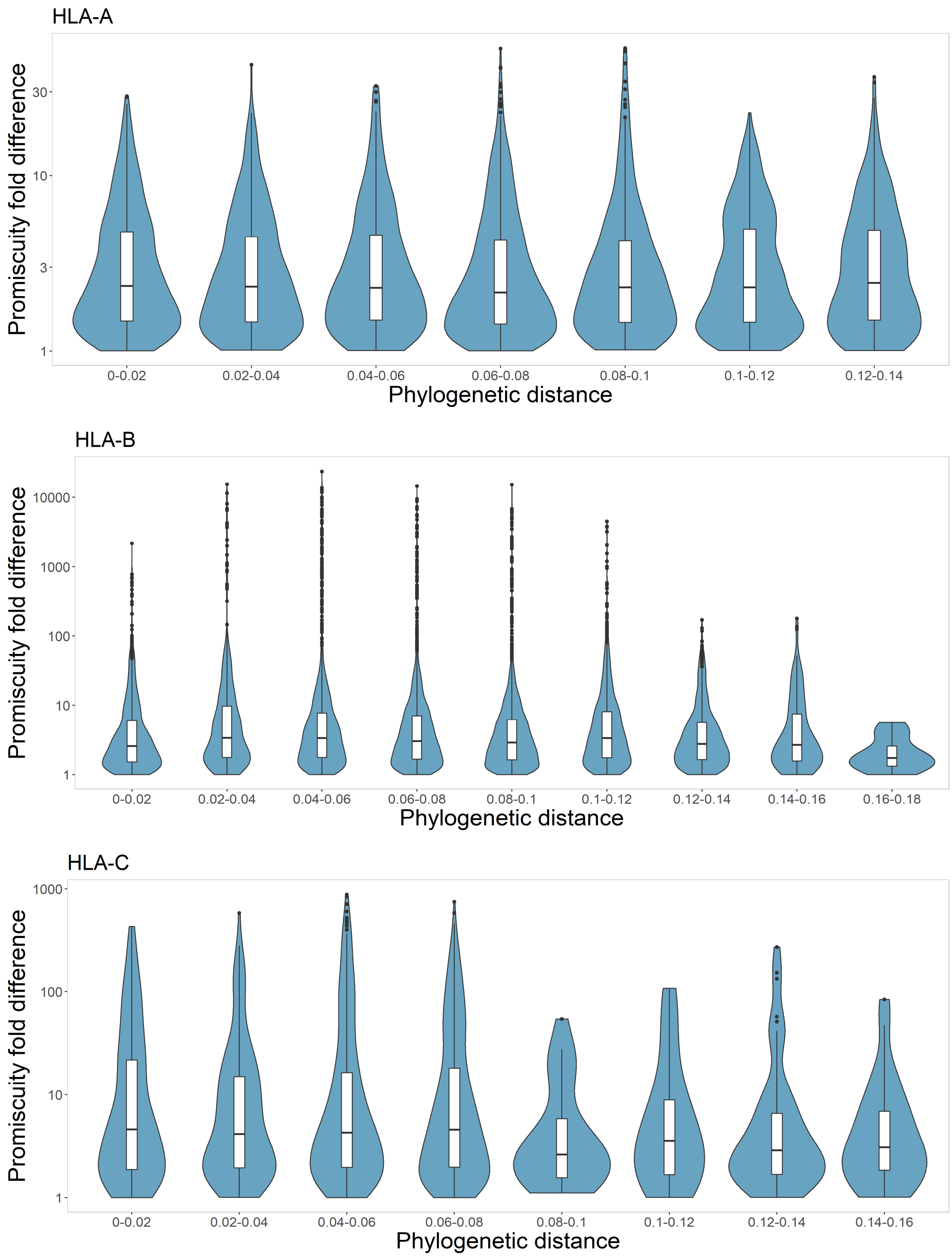

Figure S5. Differences in promiscuity in comparison to phylogenetic distance between pairs of HLA variants (HLA-A; n = 82, HLA-B; n = 180 and HLA-C; n = 59).

Phylogenetic distance between each pair is calculated as tip-to-tip distance in a phylogenetic tree. Promiscuity differences were calculated for each pair as the ratio of the number of bound peptides of more promiscuous variant to the number of bound peptides of the less promiscuous variant. Variant pairs were binned based on phylogenetic distance for better visualization.

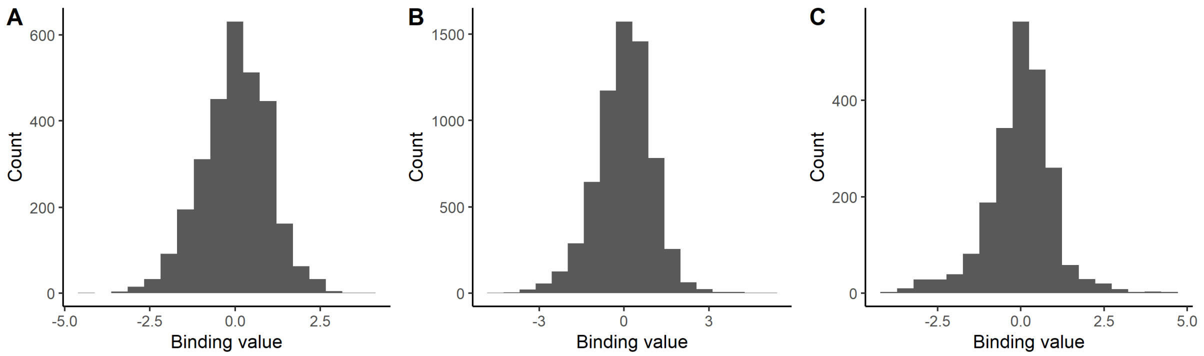

Figure S6. Distribution of standardized binding proportions for (A) HLA-A (n = 82), (B) HLA-B (n = 180) and (C) HLA-C (n = 59) loci. The fraction of bound peptides from each pathogen was normalized for each allele to obtain binding values (i.e. standardized binding proportions).

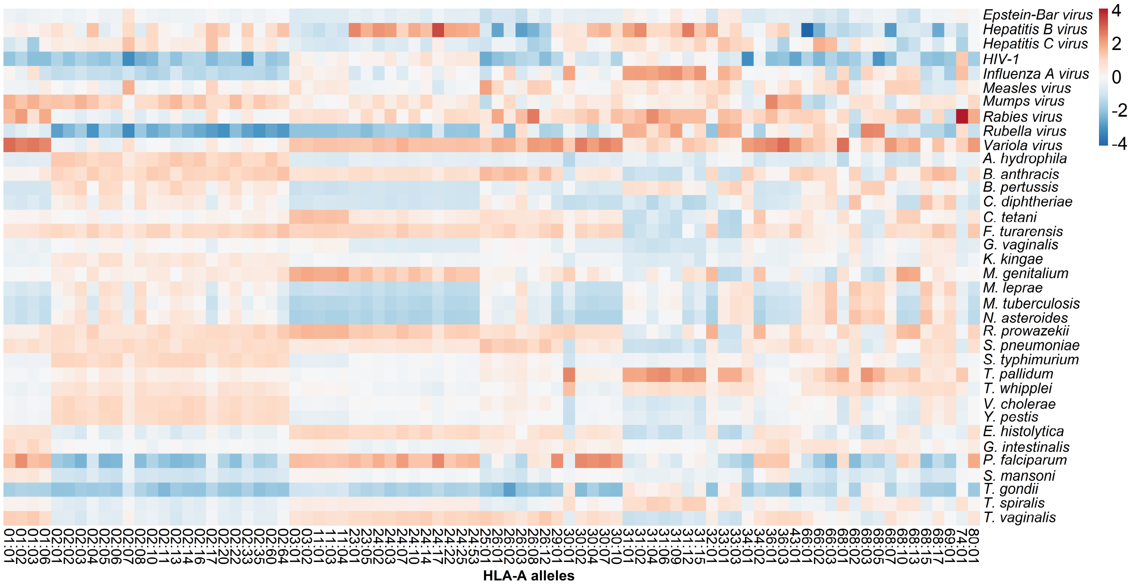
Epstein-Bar virus
Hepatitis B virus
Hepatitis C virus
HIV-1
Influenza A virus
Measles virus
Mumps virus
Rabies virus
Rubella virus
Variola virus
A. hydrophila
B. anthracis
B. pertussis
C. diphtheriae
C. tetani
F. turarensis
G. vaginalis
K. kingae
M. genitalium
M. leprae
M. tuberculosis
N. asteroides
R. prowazekii
S. pneumoniae
S. typhimurium
T. pallidum
T. whipplei
V. cholerae
Y. pestis
E. histolytica
G. intestinalis
P. falciparum
S. mansoni
T. gondii
T. spiralis
T. vaginalis
4
2
0
-2
-4
01:01 01:02 01:03 01:06 02:01 02:02 02:03 02:04 02:05 02:06 02:07 02:08 02:10 02:11 02:13 02:14 02:16 02:17 02:20 02:22 02:23 02:35 02:60 02:64 03:01 11:01 11:03 11:04 23:01 23:05 24:02 24:03 24:07 24:10 24:14 24:17 24:22 24:25 24:53 25:01 26:01 26:02 26:03 26:08 26:12 29:01 30:01 30:02 30:04 30:07 30:10 31:01 31:02 31:04 31:06 31:09 31:12 31:15 32:01 33:01 33:03 34:01 34:02 36:01 36:03 43:01 66:01 66:02 66:03 68:01 68:02 68:03 68:05 68:07 68:10 68:13 68:15 68:17 69:01 74:01 80:01
HLA-A alleles

72

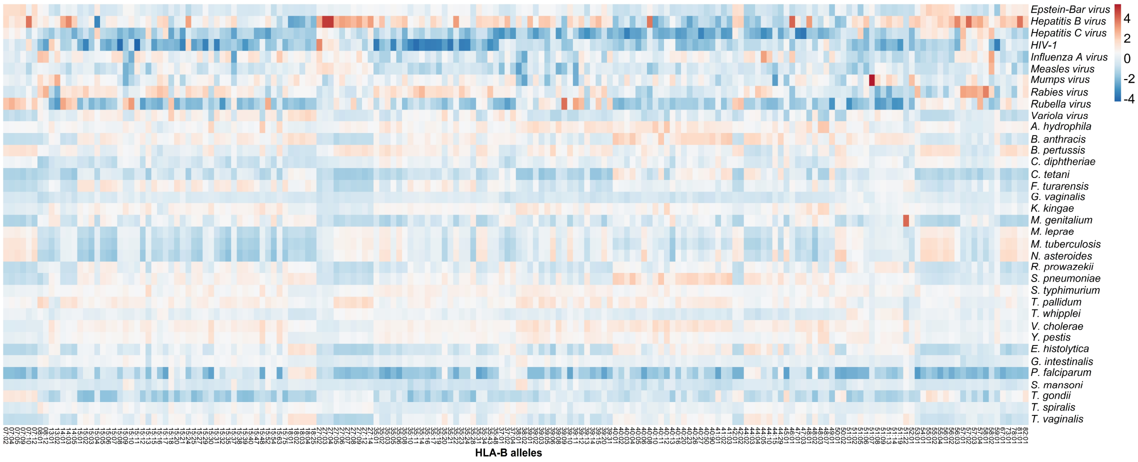
Epstein-Bar virus
Hepatitis B virus
Hepatitis C virus
HIV-1
Influenza A virus
Measles virus
Mumps virus
Rabies virus
Rubella virus
Variola virus
A. hydrophila
B. anthracis
B. pertussis
C. diphtheriae
C. tetani
F. turarensis
G. vaginalis
K. kingae
M. genitalium
M. leprae
M. tuberculosis
N. asteroides
R. prowazekii
S. pneumoniae
S. typhimurium
T. pallidum
T. whipplei
V. cholerae
Y. pestis
E. histolytica
G. intestinalis
P. falciparum
S. mansoni
T. gondii
T. spiralis
T. vaginalis
4
2
0
-2
-4
HLA-B alleles

73

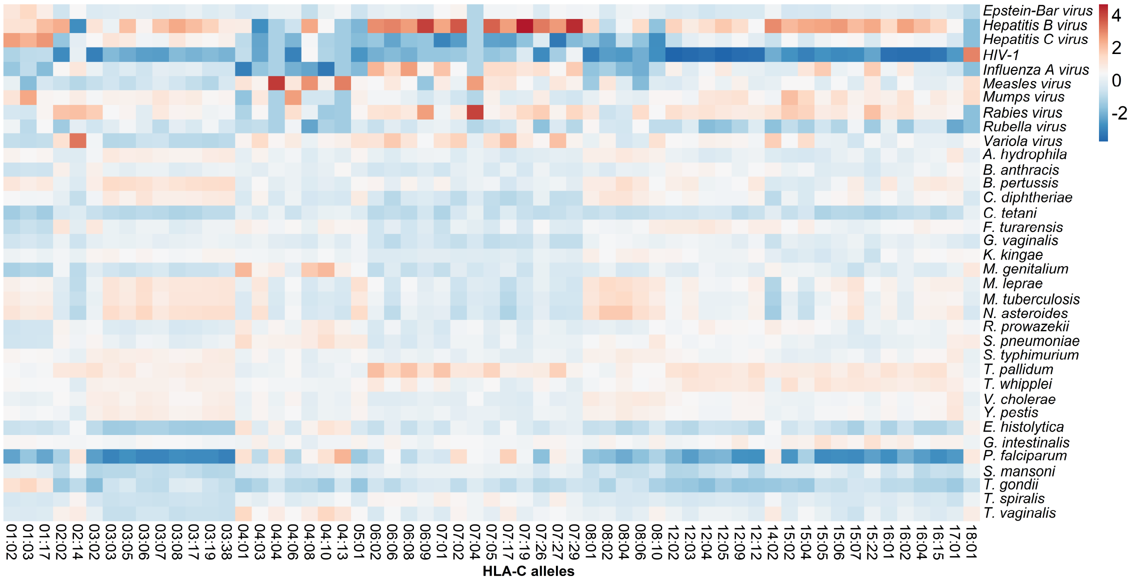
Epstein-Bar virus
Hepatitis B virus
Hepatitis C virus
HIV-1
Influenza A virus
Measles virus
Mumps virus
Rabies virus
Rubella virus
Variola virus
A. hydrophila
B. anthracis
B. pertussis
C. diphtheriae
C. tetani
F. turarensis
G. vaginalis
K. kingae
M. genitalium
M. leprae
M. tuberculosis
N. asteroides
R. prowazekii
S. pneumoniae
S. typhimurium
T. pallidum
T. whipplei
V. cholerae
Y. pestis
E. histolytica
G. intestinalis
P. falciparum
S. mansoni
T. gondii
T. spiralis
T. vaginalis
4
2
0
-2
01:02 01:03 01:17 02:02 02:14 03:02 03:03 03:05 03:06 03:07 03:08 03:17 03:19 03:38 04:01 04:03 04:04 04:06 04:08 04:10 04:13 05:01 06:02 06:06 06:08 06:09 07:01 07:02 07:04 07:05 07:17 07:19 07:26 07:27 07:29 08:01 08:02 08:04 08:06 08:10 12:02 12:03 12:04 12:05 12:09 12:12 14:02 15:02 15:04 15:05 15:06 15:07 15:22 16:01 16:02 16:04 16:15 17:01 18:01
HLA-C alleles

74

Figure S7. Standardized proportions of bound peptides by each HLA variant (x-axis) (HLA-A: n = 82; HLA-B: n = 180; HLA-C: n = 59) from each pathogen (y-axis)(n = 36). Corresponding HLA locus of each heatmap is written on the x-axis label.

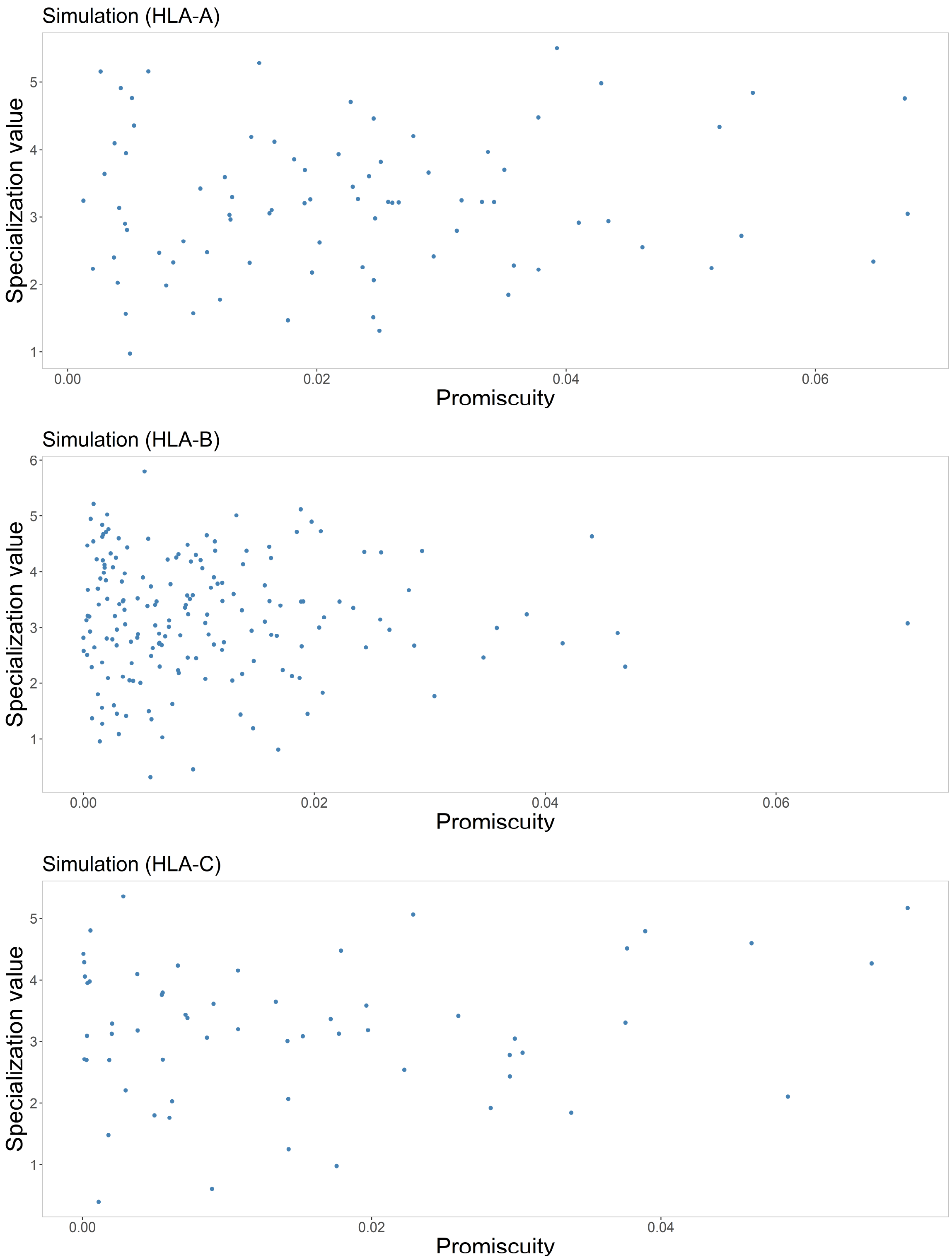

Figure S8. Specialization as a function of promiscuity in the simulated data. Each dot corresponds to a simulated HLA variant. The number of bound peptides by each HLA variant from each pathogen were simulated by using the observed promiscuity of an HLA

variant as the probability of binding a peptide. Specialization value of simulated HLA variants were calculated in the same way as the real data (i.e. difference between the maximum and the median values of standardized proportions of bound peptides). No significant correlation between specialization and promiscuity were observed for any locus in the simulated data (Kendall correlation, HLA-A: tau = 0.05 p = 0.54; HLA-B: tau = -0.02 p = 0.65; HLA-C: tau = 0.01 p = 0.93).

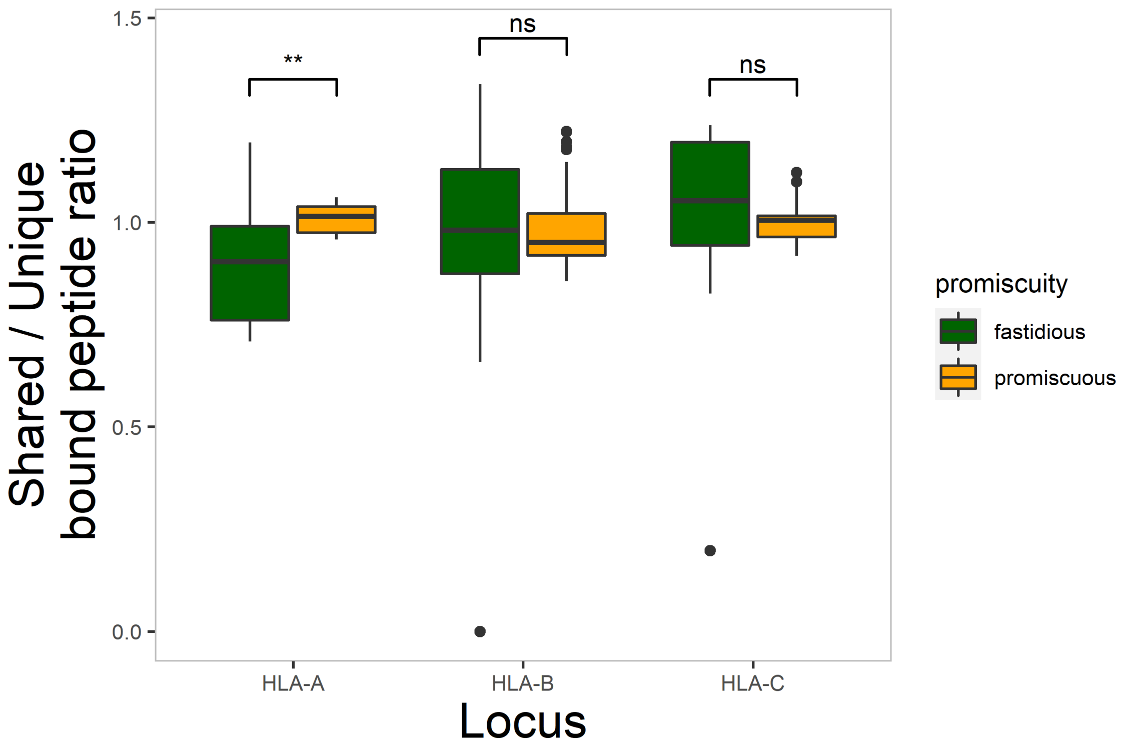

Figure S9. Comparison of the most promiscuous (top 25%) and the most fastidious (bottom 25%) HLA variants regarding binding to shared peptides. Shared peptides are the peptides observed in at least three different pathogens. The ratio on the y-axis is expected to be one if there is no tendency of alleles to bind either shared or unique peptides. ** - $p<0.01$, ns - $p>0.05$ (Wilcoxon rank sum test)

Table S1. Human pathogens used for the analysis. The asterisk (*) indicates that the pathogen is used for the pairwise peptide sharing analysis in Figure 2.

Organism	UniProt Proteome ID	Date accessed	Number of proteins	Number of nine-mers
Aeromonas hydrophila	UP000000756	11.08.2018	4121	1330710
Bacillus anthracis (*)	UP000000594	11.12.2018	5490	1375030
Bordetella pertussis (*)	UP000002676	3.07.2018	3258	1013488
Clostridium tetani (*)	UP000001412	3.07.2018	2415	780947
Corynebacterium diphtheriae (*)	UP000002198	3.07.2018	2265	701220
Entamoeba histolytica	UP000001926	3.07.2018	7959	2972321
Epstein-Barr virus	UP000153037	3.07.2018	92	39553
Francisella turarensis	UP000001174	11.08.2018	1528	459802
Gardnerella vaginalis	UP000001453	11.08.2018	1365	470742
Giardia intestinalis (*)	UP000001548	3.07.2018	7154	3048781
Hepatitis B virus	UP000007930	3.07.2018	7	1760
Hepatitis C virus	UP000000518	3.07.2018	2	3154
HIV1	UP000002241	11.12.2018	9	3062
Influenza A virus	UP000009255	3.07.2018	13	4508
Kingella kingae	UP000004207	11.08.2018	2102	551780
Measles virus	UP000008699	3.07.2018	8	4907
Mumps virus	UP000002331	11.12.2018	8	4759
Mycobacterium leprae (*)	UP000000806	11.12.2018	1603	521894
Mycobacterium tuberculosis (*)	UP000001584	12.06.2018	3993	1263777
Mycoplasma genitalium (*)	UP000000807	11.12.2018	483	172194
Nocardia asteroids	UP000017048	11.08.2018	6459	2018795
Plasmodium falciparum (*)	UP000001450	16.06.2018	5449	3806032
Rabies virus	UP000008649	3.07.2018	5	3570
Rickettsia prowazekii (*)	UP000002480	11.08.2018	834	271251
Rubella virus	UP000000571	3.07.2018	2	3162
Salmonella typhimurium (*)	UP000001014	11.08.2018	4431	1341710
Schistosoma mansoni	UP000008854	3.07.2018	11723	4939504
Streptococcus pneumonia (*)	UP000002642	2.07.2018	2823	541479
Toxoplasma gondii	UP000002226	3.07.2018	8404	6440952
Treponema pallidum (*)	UP000000811	11.08.2018	1027	337608
Trichinella spiralis	UP000006823	3.07.2018	16041	4319328
Trichomonas vaginalis	UP000001542	3.07.2018	50190	11405499
Tropheryma whpplei	UP000002200	11.08.2018	805	251906
Variola virus	UP000002060	3.07.2018	199	52593
Vibrio cholera (*)	UP000000584	2.07.2018	3783	1110752
Yersinia pestis (*)	UP000000815	3.07.2018	3909	1193604
